# Toxicity Assessment and Control of Early Blight and Stem Rot of *Solanum tuberosum* L. by Mancozeb-Loaded Chitosan–Gum Acacia Nanocomposites

**DOI:** 10.3390/jox12020008

**Published:** 2022-04-14

**Authors:** Ravinder Kumar, Joginder Singh Duhan, Anju Manuja, Pawan Kaur, Balvinder Kumar, Pardeep Kumar Sadh

**Affiliations:** 1 Department of Biotechnology, Chaudhary Devi Lal University, Sirsa 125055, India; pardeep.sadh@gmail.com; 2 ICAR-National Research Centre on Equines, Sirsa Road, Hisar 125001, India; amanuja@rediffmail.com (A.M.); bmanuja.nrce@gmail.com (B.K.); 3 TERI Deakin Nanobiotechnology Centre, The Energy and Resources Institute, New Delhi 110003, India; poonamkohar@gmail.com

**Keywords:** chitosan–gum acacia nanocomposite, ionic gelation, mancozeb, antifungal efficacy, cytotoxicity, plant diseases

## Abstract

Biopolymers such as chitosan and gum acacia are used for nanotechnological applications due to their biosafety and ecofriendly nature. The commercial fungicide mancozeb (M) was loaded into chitosan–gum acacia (CSGA) polymers to form nanocomposite (NC) CSGA-M (mancozeb-loaded) measuring 363.6 nm via the ionic gelation and polyelectrolyte complexation method. The physico-chemical study of nano CSGA-M was accomplished using dynamic light scattering (DLS), scanning electron microscope (SEM), transmission electron microscope (TEM), X-ray diffraction (XRD), and thermogravimetric analysis (TGA). Nano CSGA-M-1.0 (containing 1.0 mg/mL mancozeb) at 1.5 ppm demonstrated a maximum inhibition (83.8 ± 0.7%) against *Alternaria solani,* while *Sclerotinia sclerotiorum* exhibited a 100% inhibition at 1.0 and 1.5 ppm through the mycelium inhibition method. Commercial mancozeb showed an inhibition of 84.6 ± 0% and 100%, respectively, for both fungi. In pot house conditions, NCs were found to exhibit good antimicrobial activity. Disease control efficiency (DCE, in %) in pathogen-treated plants for CSGA-M-1.0 was 64.6 ± 5.0 and 60.2 ± 1.4% against early blight and stem rot diseases, respectively. NCs showed lower cytotoxicity than commercial mancozeb at the given concentration. In conclusion, both in vitro and in vivo antifungal efficacy for nano CSGA-M was found to be quite comparable but less toxic than mancozeb to Vero cell lines; thus, in the future, this formulation may be used for sustainable agriculture.

## 1. Introduction

The potato is an important vegetable which produced and consumed in vast quantities worldwide, and the crop benefitted the Indian economy, providing approximately INR 295 billion in 2018 [1]. The potato crop is affected by many fungal diseases in pre-harvest and post-harvest conditions. The early blight disease of potatoes is caused by two species of genus *alternaria* (*A. solani* and *A. alternata*), which are the major menaces to potato production, and are present worldwide. In India, this disease may cause up to a 40% loss in yield. Managing early blight is particularly challenging, since it produces an enormous amount of secondary inoculums [2]. Applying chemical fungicides is essential in the management of early blight disease of potatoes. Although there is no powerful bio-control method to control early blight and stem rot (caused by *S. sclerotiorum*), it is essential to improve such management tactics, since bio-control measures are unambiguous, effective, and environmentally safe [3].

Mancozeb is a freely water-soluble, non-systemic contact fungicide, which makes it an efficient fungicide, but has disadvantages in the form of introducing pollution to water bodies. Moreover, their direct application to plants presents problems in the forms of degradation by light, temperature, humidity, pH, all of which can be overcome by encapsulating the chemical fungicide in biopolymers [4]. Biocompatibility and the ability to encapsulate diverse compounds make these biopolymers ideal carriers for plant ingredients [5,6,7]. Chitosan is a natural polymer acquired from the deacetylation of chitin, and is naturally found in crustaceans (prawn, shrimp, oyster etc.), insects, and mollusks [8]. Many investigators have recognized it as a plant growth promoter and antifungal agent [9,10,11], alongside its ability to elicit defense-related enzymes in cereals and horticultural crops [12,13,14,15,16] due to its fungicidal and fungistatic potential against plant pathogens [17]. Gum acacia is a viscous, non-toxic biopolymer used in drug delivery due to its viscous nature [18]. Health and environmental concerns have encouraged authorities all over the world to consider limiting the use of or imposing a ban on many current pesticides, leading to a hunt for safe substitutes for the control of pests and other agricultural insects. Nanotechnology proves an effective means for enhancing agronomic potentials [19,20,21,22,23]. Nanotechnology uses the most negligible concentration and utmost in pest control to decrease the cost of pest control due to its small size [24,25]. The nanoencapsulation of fungicide in biopolymers, such as chitosan and gum acacia, has been applied to commercial fungicides, and is capable of enhanced disease potency due to controlled release, site-directed delivery, and reduced toxicity [23,26,27,28,29,30,31,32].

Thus, the goal of present investigation was, first, to form mancozeb-loaded nanocomposites (NCs) consisting of chitosan–gum acacia, and to assess their in vitro and in vivo antifungal ability on fungal pathogens of potato crops. Second, we determined the cytotoxicity of these nanocomposites to Vero cells.

## 2. Materials and Methods

### 2.1. Materials and Microorganisms

Chitosan (CS) and sodium tripolyphosphate (TPP) were procured from Sigma-Aldrich, USA, and gum acacia (GA) from Qualigen, India. Commercial mancozeb was obtained from the local market. The in vivo pot experiment was performed on potato variety Kufri Pukhraj (procured from Vegetable Division, CCSHAU, Hisar, India) in a greenhouse at 25 °C. The National Research Center on Equines, Hisar, maintained the Vero cell line. Potato pathogenic fungi (*A. solani* and *S. sclerotiorum*) were procured from Indian Type Culture Collection, IARI, New Delhi, and were revived for purifications.

### 2.2. Synthesis of Chitosan–Gum Acacia Nanocomposites

Conjugated nanocomposites were synthesized via the ionic gelation and polyelectrolyte complexation methods [33]. For this, a 1.0 mg/mL stock solution of chitosan (pH 5) was prepared in 1% glacial acetic acid (*v*/*v*) by constant stirring to dissolve completely. A certain amount of mancozeb powder was weighted in three separate flasks, and the stock solution of CS as prepared above was added to each flask. Then, aqueous gum acacia (1.0 mg/mL; in double-distilled water, pH 5) was added under continuous stirring to each flask to produce final concentrations of mancozeb at 0.5, 1.0, and 1.5 mg/mL in respective flasks. Then, 2.0 mL of 1%TPP was dropwise dispensed to the above solution with continuous stirring for 45 min. It was followed by the addition of Tween-20 (a surfactant) to prevent the aggregation of NCs. The suspension was centrifuged at 12,000 rpm for 20 min, and the pellet was washed twice with double-distilled H_2_O; the pellet obtained was then oven-dried.

For the synthesis of blank NCs, the addition of mancozeb was skipped in the above procedure.

### 2.3. Size, Polydispersity Index (PDI) and Zeta Potential

NCs were dispensed in a non-reusable cuvette and disseminated in 950 µL double distilled water, and graph peaks were obtained at 25 °C using Nano ZS90 (Malvern Instrumentations, Holtsville, UK).

### 2.4. Transmission Electron Microscopy (TEM)

Technai^TM^ (Thermo Fisher Scientific, Waltham, MA, USA) was used for air dried samples on carbon-coated copper grid. The images were obtained at 200 kV at different magnifications.

### 2.5. Scanning Electron Microscopy (SEM)

SEM micrographs were obtained by a 10 mg dry powder sample on an aluminum stub along with carbon pasted disc coated with gold through JSM-6390LV (JEOL Ltd., Tokyo, Japan).

### 2.6. X-ray Diffraction Spectroscopy (XRD)

The powder sample was tested in a step scan mode with a tube voltage of 40 kV on a D-8 Advance Diffractometer (Bruker AXS, Karlsruhe, Germany). The samples were scanned in the 2θ range of 5 to 40° at a current flow of 40 mA.

### 2.7. Thermogravimetric Analysis (TGA)

TGA was performed at CIF, LPU, Jalandhar, using a Thermo Gravimetric Analyzer (TGA 4000, Perkin Elmer, Billerica, MA, USA). The instrument was heated at a rate of 10 °C/min from 30 to 445 °C, then held at 445 °C for 1 min. To maintain an inert environment during the measurement, pure nitrogen gas was injected into the apparatus at a rate of 20 mL/min.

### 2.8. Antimicrobial Activity Using Mycelium-Inhibition Method

The antifungal activity was determined using the mycelium-inhibition technique. Different quantities of nanocomposites (0.5, 1.0, and 1.5 ppm) were introduced to an autoclaved potato dextrose agar medium, which was then placed onto sterile Petri plates and allowed to harden. A mycelial disc of uniform size (5.0 mm in diameter) from a seven-day-old test pathogen culture was put in the middle of test Petri plates, and incubated at 28 ± 1.0 °C. The experiment was repeated three times. After four days, the radial mycelial growth was measured. The mycelia percentage suppression by nanocomposites was calculated by comparing the infected plates to the control (without nanocomposites) using the formula:

Percent inhibition = dc − dt/dc × 100

where dc is the mycelial growth diameter in control, and dt is the mycelial growth diameter in treatment.

### 2.9. Vero Cell Culture and Toxicity Testing

Using resazurin dye (Hi-Media Pvt. Ltd., Mumbai, India) as a colorimetric technique, the cytotoxic activity of nanocomposites was determined. The resazurin test procedure is based on the mitochondrial respiratory chain converting oxidised resazurin (non-fluorescent blue) to a red fluorescent dye (resorufin) in living cells. Since dead cells have a high absorbance, cell viability/cytotoxicity was determined with the use of absorbance [34].

In a 96-well cell culture plate, Vero cell lines (from the African green monkey) were cultivated at a density of 1 × 10^4^ per well in a 100 µL volume of Eagle’s minimum essential medium (EMEM, 10% foetal bovine serum, and antibiotics added). The cultivated cells were treated with varied concentrations of nanocomposites (0.03 µg/mL to 2000 µg/mL), thoroughly dispersed in 100 µL of deionized water via sonication, and incubated for 24 h in a CO_2_ incubator (at 37 °C with 5% CO_2_). Following incubation, the samples were treated with 10 µL of resazurin dye (1.0 mg/mL, prepared in EMEM medium) and incubated under the same conditions for 4 h. The pink-colored resorufin was generated after 4 h due to mitochondrial activity, and the ELISA plate reader (Biotek Instruments, Winooski, VT, USA) detected absorbance at 590 nm.

### 2.10. In Vivo Pot House Experiment

Seeds were treated for 10 min with 4% sodium hypochlorite (a surface disinfectant), and then rinsed twice with distilled water. Seeds were then soaked for 10 min in carboxymethyl cellulose (CMC, 5.0 gm in 100 mL distilled water) and dried. Nanocomposites (10 ppm) were applied to the seedlings for 2 h and 30 min, after which they were allowed to dry at room temperature. In total, three seeds/pots were seeded in pots filled with soil (pH 7.7 at 20 °C) that had previously been infected with pathogenic fungus two days before sowing, and they were watered regularly [22].

To induce the illness, 40-day-old plants were sprayed (15 mL/plant) with an aqueous conidial solution (3.1 × 107 CFU/mL) of the various pathogens. To maintain the humidity essential for disease outbreak, the plants were covered with clear plastic bags. After seven days of conidial pathogen spray, disease severity (DS, in percent) was measured, and a foliar spray of polymeric NCs (10 ppm, 10 mL/pot) was applied to test the disease control effectiveness (DCE, in percent) of NCs against the disease outbreak. As a positive control, the commercial fungicide mancozeb was employed.

The disease severity percentage (DS percent) was calculated at random using the conventional 0–5 scale [13].

Disease severity (DS, %) = (Sum of all individual disease ratings)/(Total number of plants examined − maximum rating) × 100

The Kaur et al., 2018 [23] algorithm was used to compute disease control effectiveness (DCE, in percent).

Disease control efficacy (DCE, %) = (Disease severity in control − Disease severity in treatment/(Disease severity in control) × 100

To evaluate the overall health and vigor of plants following NCs treatment, bio efficacy was measured in terms of plant growth metrics such as plant height, root-shoot ratio, and plant dry weight.

### 2.11. Statistical Analysis

All of the studies were performed in triplicate, and the findings were provided as mean ± std dev (SD). Microsoft Office Excel 2013 was used to process statistical data (Microsoft Corporation, Albuquerque, NM, USA). The one-way analysis of variance was used to assess statistical differences between sets. A *t*-test was used to determine statistical significance at a level of *p* ≤ 0.05.

## 3. Results and Discussion

### 3.1. Nanocomposites Synthesis, and Particle Size Analysis

**Figure 1:**
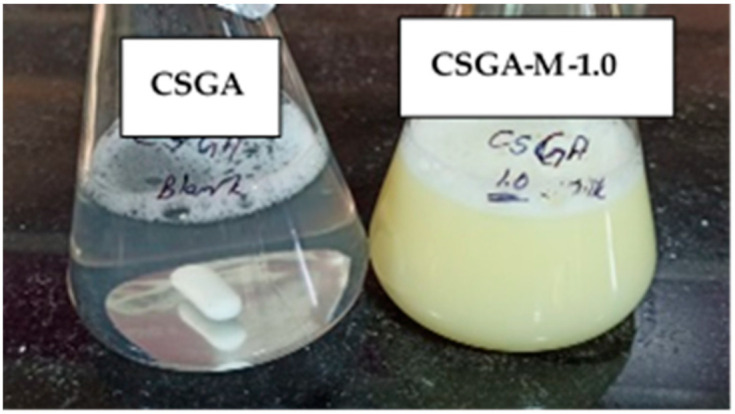
Blank and mancozeb-loaded CSGA nanocomposites.

**Figure 2:**
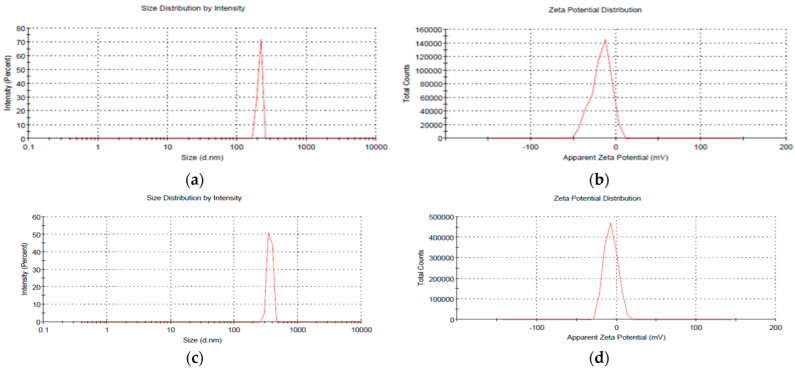
(**a**) Particle size and (**b**) zeta potential of CSGA blank NCs; (**c**) particle size and (**d**) zeta potential of nano CSGA-M-1.0 (CSGA NCs containing 1.0 mg/mL mancozeb).

The middling size of 211.8 nm was recorded in blank NCs (i.e., without mancozeb loading), with a 1.00 PDI and zeta potential of −17.1 mV, but the size of 363.6 nm was reported in NCs with 1.0 mg/mL mancozeb (nano CSGA-M-1.0) nanoformulation (Figure 1 and Figure 2a–d and Table 1).

The loading of mancozeb enhanced the size of polymeric NCs. Many parameters influence the size of NPs, including chitosan molecular weight and deacetylation %, pH, stirring speed and duration, and a reverse micellar approach yields stable, tiny, uniform-sized chitosan nanoparticulates [35]. Higher pH was also observed to reduce the mean size, polydispersity index, and zeta potential of chitosan nanoparticles, owing to constricted chitosan chains, and hence improved hydrogen bonding [36]. In previous research, 450–500 nm chitosan-carrageenan nanoparticles with a zeta potential of 75–85 mV were seen [37]. Another study created metsulfuron-methyl (algrip)-loaded pectin nanocapsules with a zeta potential of −35.9 mV in the size range of 50–90 nm [29]. The current study’s size and zeta potential are consistent with the previously mentioned literature.

### 3.2. Transmission Electron Microscope/Scanning Electron Microscope (TEM/SEM)

**Figure 3:**
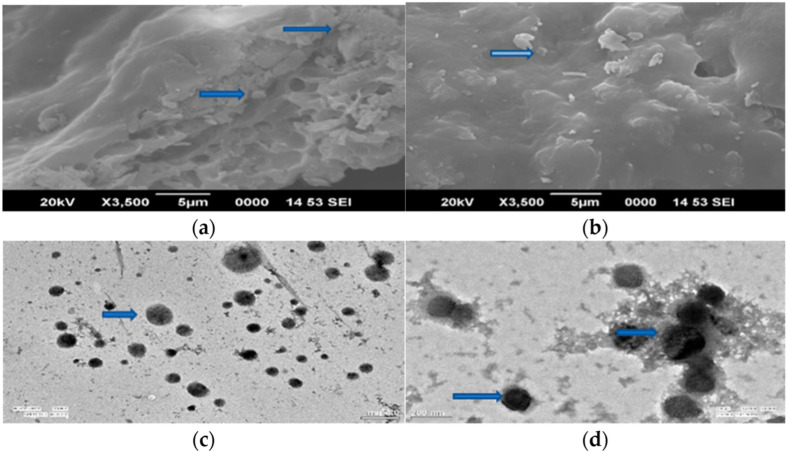
SEM and TEM micrographs of blank and mancozeb loaded NCs: SEM (**a**) blank CSGA NCs; (**b**) CSGA-M-1.0: NCs containing 1.0 mg/mL mancozeb; TEM: (**c**) Blank CSGA NCs; (**d**) NCs containing mancozeb i.e., CSGA-M-1.0.

At 3500X magnification, SEM micrographs of blank NCs revealed spherical shaped NCs with hollow interiors; however, no such hollow structures were discovered in mancozeb-loaded NCs (Figure 3a,b). The synthesis of well-dispersed, small-sized, round-shaped nanocomposites was validated by TEM data, which revealed mancozeb within the NCs as a dark grey patch and a fiber-like network connecting the NCs (Figure 3c,d, blue arrow). Solid, dense NPs were detected in acetamiprid-loaded alginate-chitosan nanocapsules which were coupled to one another in a similar work [38].

The size achieved in this investigation matches the size and shape seen in earlier SEM and TEM studies. A TEM image, for example, revealed solid dense nanocapsules of 30 to 40 nm that were attached to one another [38]. In a TEM micrograph of hexaconazole-loaded chitosan nanoparticles, spherical shaped nanocapsules of less than 100 nm were described [4]. Another researcher discovered 150 nm formulations while dealing with Cu-chitosan NPs. Previously, very porous structures were seen at greater magnification in SEM pictures, as observed in the current work [27]. Another study found 70–90 nm particles in TEM pictures of carbendazim-loaded pectin–chitosan nanoparticles, with a count of 15–20 particles in the microscope [29].

### 3.3. Thermogravemetric Ananlysis (TGA)

**Figure 4:**
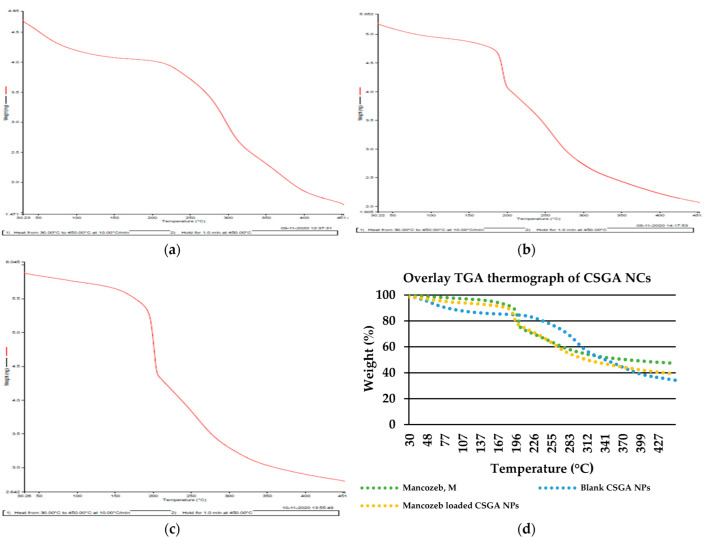
Thermogravimetric (TGA) thermograms of (**a**) CSGA blank NCs; (**b**) CSGA-M-1.0 NCs; (**c**) commercial mancozeb; in the form of weight, in mg; (**d**) overlay TGA thermograph in the form of weight %.

The breakdown of mancozeb occurred in two stages, each with its own set of gases. Between 30 °C and 200 °C, the first weight loss occurred. The weight of mancozeb was lowered to 4.4 mg from 6.6 mg (a 24.1 percent weight drop), and is mostly associated with CS_2_ and H_2_S emissions, along with minor quantities of CO and SO_2_. The second weight loss (21.4%), caused by H_2_S emissions, occurred between 200 and 300 °C [39]. At around 190 °C, commercial mancozeb shows a significant weight loss, indicating that it is decomposing. Blank and mancozeb-loaded CSGA NCs showed no such dramatic weight loss, indicating that they are more thermally stable than commercial mancozeb (Figure 4a–d).

Previously, TGA data revealed that altering alginate microbeads with chitosan and carrageenan increased the thermal strength of alginate microbeads [40]. The initial weight reduction in blank CSGA NCs at 30–200 °C may be attributed to water loss. Weight loss at around 200 °C might be ascribed to CSGA NC disintegration, in which mancozeb and CSGA NCs dissolve simultaneously [41]. These findings support a prior work in which the TGA curve of diclofenac sodium-loaded chitosan/carrageenan revealed that the third stage (232–310 °C) was caused by simultaneous drug and polymer degradation [42].

### 3.4. X-ray Diffraction Spectroscopy (XRD)

**Figure 5:**
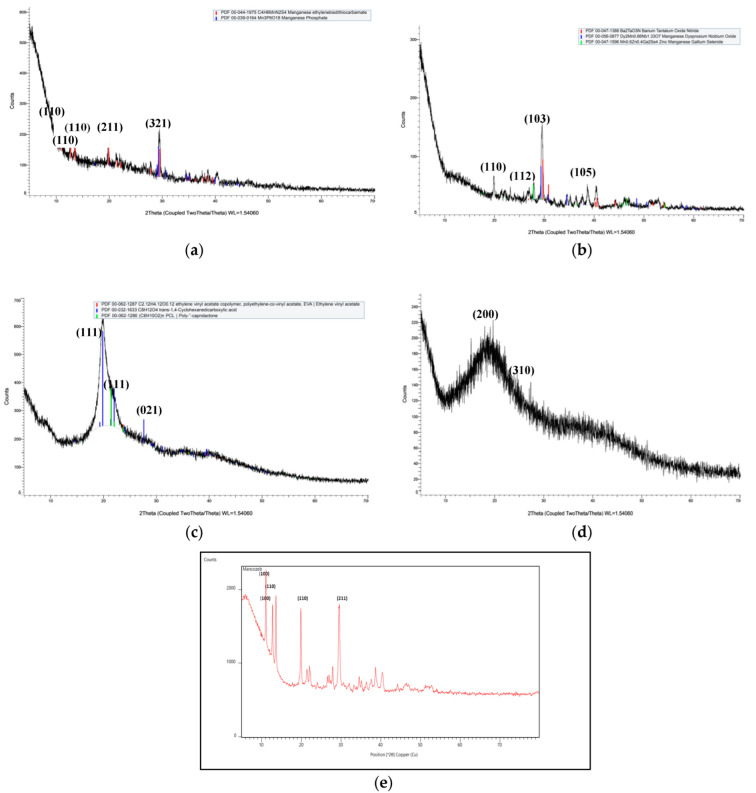
XRD peaks of blank and mancozeb loaded nanoformulations: (**a**) chitosan–gum acacia blank nanocomposites; (**b**) mancozeb loaded chitosan–gum acacia nanocomposites; (**c**) chitosan biopolymer; (**d**) gum acacia biopolymer; (**e**) mancozeb.

As shown in Figure 5e, strong peaks at 2θ values were found in mancozeb at 11.0272°, 12.7212°, 13.5734°, 19.8453°, and 29.5710°, which showed highly crystalline nature of mancozeb and Miller indices (h k l) for these peaks at 100, 100, 110, 110, and 211, respectively. Strong peaks at 2θ values were found for chitosan–gum acacia nanoformulations at 11.079°, 12.74°, 13.59°, 19.89°, and 29.55°, which showed the crystalline nature of NCs and Miller indices (h k l) values for these peaks were 110, 110, 110, 211, and 321, respectively (Figure 5a). A broad peak is seen in the chitosan polymer at 20°, which confirms the semi-crystalline nature of polymer. For chitosan–gum acacia nanocomposites containing mancozeb, a sharp peak at 29.5710° is observed, which is also seen in commercial mancozeb, and the crystalline peak of mancozeb was buried underneath when mancozeb was encapsulated in chitosan–gum acacia nanocomposites.

This study’s XRD pattern is similar to that of another scientist’s work, in which a strong peak of dazomet was noticed and buried down when contained in chitosan nanoparticles [35]. Furthermore, the strong peaks of mancozeb loaded nanocomposites at the diffraction angles of 19°, 29°, 38°, and 41° matched the peak pattern of commercial mancozeb, indicating that mancozeb is encapsulated in chitosan–gum acacia (CSGA) nanocomposites (Figure 5b,e).

### 3.5. In Vitro Antifungal Activity

**Figure 6:**
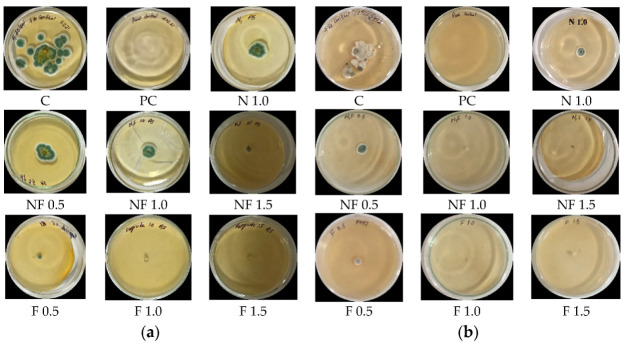
In vitro mycelium inhibition efficacy of CSGA NCs at three concentrations (0.5, 1.0, and 1.5 ppm) against potato pathogens: (**a**) *A. solani* (**b**) *S. sclerotiorum*; where: C—control, PC—pure control (PDA alone), N 1.0-blank CSGA NCs at 1.0 ppm, NF—fungicide loaded CSGA NCs, F—commercial fungicide.

In the case of *Alternaria solani*, the maximum inhibition (83.8 ± 0.7%) was observed in mancozeb-loaded nanoformulation at 1.5 ppm. The commercial mancozeb at this concentration had an inhibition of 84.6 ± 0%, therefore it can be said that the inhibition exhibited by NCs is fairly equivalent to marketable fungicide at the same dose. A complete inhibition was observed for mancozeb-loaded NCs at 1.0 and 1.5 ppm against *S. sclerotiorum,* and is statistically significant, according to the *t*-test. Outcomes in current experiment are in agreement with an earlier study, and are quite comparable where 15 ppm concentration caused a 99.6% reduction in mycelial growth over the control in *M. phaseolina,* and thorough inhibition (100% reduction) was achieved at 25 ppm via the poisoned medium technique [29]. The percentage inhibition exhibited by nano CSGA-M against potato pathogens is shown in Figure 6a,b and Table 2. In earlier research, the inhibitory effect of NPs was prompted by the small particle size of chitosan nanoparticles, as compared to their bulky counterpart [43], and is also proved in the present research. Earlier, fungicidal potential of nanohexaconazole was also found to be better, as compared to its conventional form. The respective percentage fungal growth inhibitions at 0.01 to 0.1 ppm were found to be in the range of 18.8–71.1 and 9.3–41.8% for nano and commercial hexaconazole, respectively [38].

Chitosan has well documented antifungal activity, and in earlier research, low molecular weight chitosan alone or in combination with the commercial mancozeb controlled the spore formation for late blight and wilt caused by the pathogens *P. infestans* and *Fusarium solani* f. sp. *eumartii*, respectively [44]. Increasing the concentration of the nanofungicide provided better results, and was comparable to non-nanoform results. Another researcher loaded azoxystrobin, pyraclostrobin, tebuconazole, and boscalid in lignin NPs to control *Phaeomoniella chlamydospora* and *Phaeoacremonium minimum* fungi (causative agent of grapevine trunk disease), which was achieved after 96 h in vitro conditions [45]. In the another study, in vitro effectiveness experiments demonstrated that 0.1 percent Cu-chitosan NPs inhibited mycelial development by up to 98 percent against *Rhizoctonia solani* and *Pythium aphanidermatum, which* cause damping-off disease. [46]. In the same manner, fungicide hexaconazole-loaded chitosan NPs provided better inhibition of *Ganoderma boninense,* with lower EC_50_ values to fight Ganoderma disease in oil palm plants [35]. The current study’s findings are consistent with previous findings, and have the potential to considerably improve the productivity of the world’s most important and commonly farmed vegetable crop, the potato, by reducing fungal infections.

### 3.6. In Vivo Antifungal Efficacy

**Figure 7:**
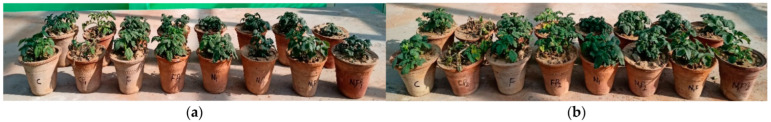
Efficacy of CSGA NCs in pot s against potato pathogens (**a**) *Alternaria solani—*P1, (**b**) *Stemphylium lycopersici—*P2; where C—Control, CP1—pathogen P1, F—commercial fungicide, FP1—commercial fungicide + P1, N1—blank NCs, N1P1—pathogen P1 + blank NCs, N1F—mancozeb loaded NCs, N1FP1—loaded NCs + P1; CP2—pathogen P2, FP2—fungicide + P2, N1P2—blank NCs + P2, N1FP2—mancozeb-loaded NCs + P2; P1 stands for *A. solani* and P2 for *S. sclerotiorum*.

NCs treated seeds (three seeds/pot) were allowed to germinate after conidial broth spray; symptoms of early blight and stem rot were seen in potato plants treated with the pathogen alone, whereas no such symptoms were observed in plants treated with nanocomposites. The disease efficacy of NCs is shown in Table 3 and Figure 7a,b in the form of disease severity percentage (DS%) and disease control efficacy percentage (DCE%) after ten days of foliar spray of NCs.

From Table 3, it can be observed that, after conidial spray of pathogenic fungi, the control plants developed a disease severity (DS) of 29.4 ± 1.6% for early blight and 27.4 ± 1.6% for stem rot disease. Disease control efficiency (DCE, in %) in pathogen-treated plants for loaded NCs (NFP) was found to be 64.6 ± 5.0% and 60.2 ± 1.4% against early blight (Figure 7a) and stem rot diseases (Figure 7b), respectively, which was quite comparable to pathogen-sick plants treated with commercial fungicide, FP (66.3 ± 2.2% and 52.9 ± 3.4%, respectively) plants. Thus, the study showed the efficiency of synthesized nano CSGA-M-1.0 NCs in controlling early blight and stem rot in potato plants. Similarly, another researcher managed wilt disease of chickpea in vivo via Ag NPs biosynthesized by rhizospheric microflora of *Cicer arietinum.* Chitosan–silver and chitosan NPs saw a 33.33% reduction in wilt incidence, and were least effective [13].

Pyraclostrobin, a limited water mixable fungicide, was inserted into an inchitosan-lactide copolymer to form nanofungicide, and was similar to commercial pyraclostrobin in preventing inhibition of *C*. *gossypii* after three days post-application [47]. In another study, kaempferol (a lesser-soluble fungicide put onto lecithin/chitosan) showed 67 % inhibition efficiency for *Fusarium oxysporum* [48]. Similarly, a 74.5% reduction in basal stem rot disease of palm tree [49] and chitosan NPs in wheat Fusarium head blight [50], as well as the same pattern of disease efficacy was obtained in present study.

The present results are in accordance to a previous study where, despite 1/5th of pesticide concentrations being present in NPs, the LC_50_ (lethal concentration) in NPs were found to be significantly low (2.9 and 15.9 ppm), compared to 34.3 and 66.5 ppm in two conventional pesticide formulation, respectively [51]. From the above discussion, it is evident that nano CSGA has antifungal activity which is as good as a commercial fungicide, and other growth parameters, such as germination percentage, dry biomass, and root–shoot ratio of plants, were also observed in this study.

#### 3.6.1. Effect of Treatment on Germination Percentage

**Figure 8:**
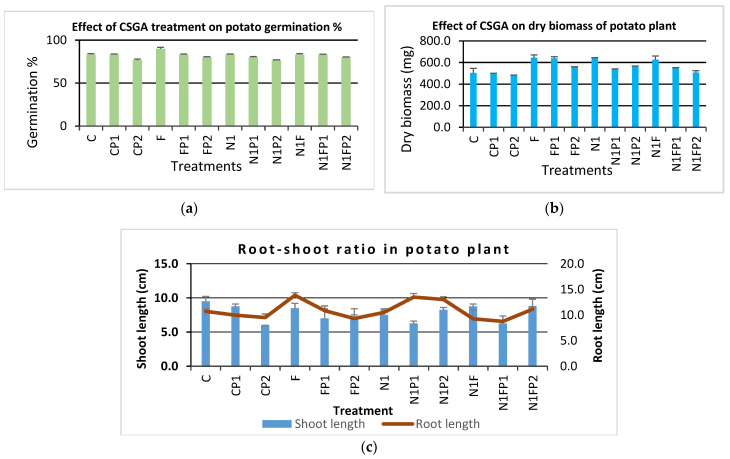
Effects of NCs on potato plant development characteristics: (**a**) germination percent; (**b**) dry biomass per plant; (**c**) root-shoot ratio; where C—Control, CP1—pathogen P1, F—commercial fungicide, FP1—commercial fungicide + P1, N1—blank NCs, N1P1—pathogen P1 + blank NCs, N1F—mancozeb-loaded NCs, N1FP1—loaded NCs + P1; CP2—pathogen P2, FP2—fungicide + P2, N1P2—blank NCs + P2, N1FP2—mancozeb-loaded NCs + P2; P1 stands for *A. solani* and P2 for *S. sclerotiorum*.

The germination data were recorded from each pot after 28 days of sowing. It is apparent from Figure 8a that the lowest germination (77% and 80%) was observed in plants diseased with *S. sclerotiorum* (CP2) and sick plants treated with mancozeb-loaded NCs (N1FP2), respectively, which is comparable to the control plants (C). Nanoparticles show varying effects on seed germination of various plants [52]. The highest germination (90% in each group) was recorded in plants treated with commercial fungicide alone. Around 80.0% germination was recorded in all other treatments, which is quite good.

There was some decline in germination percentage in pathogen-treated plants, as compared to the control, which may be due to pathogens incubated in the soil, and requires further study in terms of plant fruit biomass and yield. Earlier studies established that the nano-formulated carbendazim with a polymer was safer for the germination and root growth of *C. sativa*, *Zea mays*, and *L. esculentum* seeds, where NPs had 96% germination rates, while pure carbendazim showed 60% germination rates [29]. Similarly, wheat (*Triticum aestivum* L.) plants root length, number and mass of roots, hypocotyl length and mass, and germination rate were not significantly changed by gadoliniumorthovanadates doped with europium (GdVO4:Eu^3^) NPs at concentrations of 0, 10, 50, and 100 µg/mL dose under in vitro conditions [53].

#### 3.6.2. Potato Dry Biomass Per Plant

The dry weight of potato per plant was obtained by putting the whole plant with the roots (tuber, stolon, and parent tuber detached) in a brown envelope (three plants/envelope), and dried in a hot air oven at 40 °C for seven days. In earlier research, it was found that nanoparticles may lead to leaf discoloration and invasion, stunting, and root growth inhibition [54]. The highest mass of 643.3 mg was obtained for N1 (plants treated with blank NCs), followed by 626.7 mg for plants treated with fungicide-loaded NCs (N1F). The lowest dry biomass (500 and 481.7 mg) was obtained for plants infected with pathogens alone, respectively, while treatment increased the biomass to 546.7 and 506.7 mg, respectively, for both pathogens, and thus confirms the overall development of treated plants (Figure 8b).

#### 3.6.3. Root-Shoot Ratio

Sometimes, the treatment significantly affects root–shoot length due to mobilization of NPs in these parts, besides the disease efficacy. The plant root–shoot length was measured, starting from the root–shoot junction with a 30 cm scale just before harvesting. NP-treated plants tend to show enhanced shoot length, as compared to untreated plants, when considering pathogen *S. sclerotiorum* (6.0 cm), whereas improved or longer root lengths were seen in blank nanoformulation-treated plants which were infected with pathogens; NIP1 and N1P2 (13.5 and 13.0 cm, respectively), and requires further study at molecular levels for this pattern. Earlier, root elongation was seen with silver NPs, as compared to the control, and a drastic decrease in shoot length was observed [55], whereas silver NPs showed adverse effects on seed germinations, root, and shoot growth in *Oryza sativa*, *Vigna radiata,* and *Brassica campestris* plants at different concentrations of nanoparticles [56]. Ag NPs also weakened root and shoot growth in *Triticum aestivum* seeds [57]. In pathogen (P2, *S. sclerotiorum*)-treated plants with blank NPs, N1P1 (where shoot length was 8.3 cm), and infected plants treated with fungicide-loaded NCs (8.8 cm) had the enhanced shoot length, respectively. This proves that NC treatment enhanced the shoot length for *S. sclerotiorum,* which must be due to enhanced disease efficacy of CSGA NCs against the stem rot disease of potato crops (Figure 8c).

The use of carbon nanotubes showed various benefits on tomato crops [58]. Knowledge of nanoparticle root absorption and root-to-shoot transport may contribute to the application of nanotechnology in plant nutrition, Zn uptake, biotransformation, and *Phaseolus vulgaris* physiological impacts [59]. Near-edge XRD revealed that roots dissolved 40 nm ZnO NPs more easily than 300 nm ZnO NPs. It also revealed that Zn was detected in the leaves as a combination of Zn_3_(PO_4_)^2^ and Zn-histidine complex. The lower stem tissue segment acted as a buffer below 100 mg Zn/L, preventing Zn from reaching the leaves [60].

In conclusion, we can say that an overall positive effect of nano CSGA was observed in treated plants, as compared to control plants, for antifungal efficacy/growth parameters, and is in accordance with the results obtained by previous researchers [61,62].

### 3.7. Cytotoxicity of Nanoformulations on Vero Cell Culture

**Figure 9:**
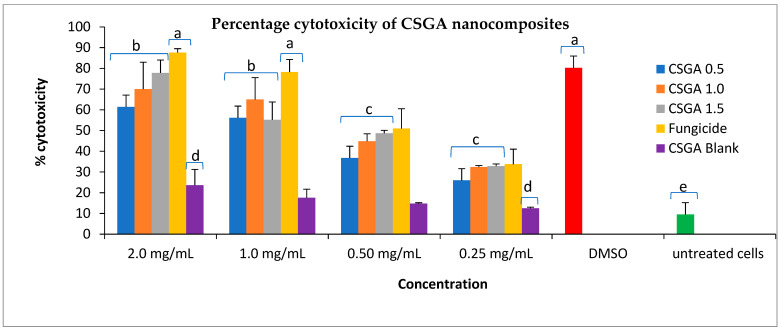
Percentage cytotoxicity of CSGA nanocomposites against the Vero cell line. Caps on bars represent SD. Same letters in graph column of each treatment are not significantly different at *p* ≤ 0.05, according to the *t*-test.

The toxicity of blank and mancozeb-loaded (0.5, 1.0 and 1.5 mg/mL) nanoformulation was tested at a concentration of 2.0 mg/mL (highest) to 0.25 mg/mL (lowest) of nano CSGA. DMSO (dimethyl sulfoxide, 10 µM)) was taken as a reference drug, and is known to be harmful to Vero cells. Untreated cells were taken as a negative control. CSGA-M-0.5 (NCs having 0.5 mg/mL mancozeb) at a concentration of 0.25 mg/mL showed the lowest cytotoxicity (25.92%), which is relatively lower than the commercial mancozeb (33.72%). Similarly, at a concentration of 2.0 mg/mL, all three mancozeb-loaded nanoformulations (i.e., CSGA-M-0.5, 1.0 and 1.5) exhibited cytotoxicity of 61.40, 69.99, and 77.78%, respectively, which is significantly lower, as compared to commercial mancozeb (87.61%). The blank NCs showed very low toxicity at all concentrations (for example, 14.71% at 0.50 mg/mL), which proves that the cytotoxicity was due to mancozeb and not due to nanocomposites [63]. The mancozeb-loaded NCs were found to be significant (shown with different letters; b and c), as compared to the blank NCs (violet, shown with letter d) and control (represented with letter; a) at *p* ≤ 0.05 in a *t*-test, whereas at lower concentrations (0.25 and 0.50 mg/mL), loaded NCs were not significantly different to each other (same letters; c). The cytotoxicity decreased with a decrease in NCs concentration for all three formulations, as shown in Figure 9. The results of this study are in accordance with a previous study, and thus validated, where fungicide-loaded chitosan–carrageenan nanoparticles exhibited almost same IC_50_ (inhibitory concentration 50) at 0.50 mg/mL against Vero cells using a Resazurin assay [63].

The reduction in toxicity of mancozeb-loaded nanoformulation may be due to the slow and sustained release of mancozeb from the CSGA nanomatrix [40,64,65]. Similarly, the encapsulated herbicide paraquat was found to be less toxic to alveolar and mouth cell lines (A549), as compared against the trade form. [42]. An earlier study showed that the application of herbicide-loaded nanoparticles could be used to reduce the use of herbicides with improved efficacy and environmental safety [29]. While working with sodium alginate nanoparticles on cucumber plants, leaching of commercial herbicide tebuthiuron in soil was found to be higher (40–50 cm deep), while for nanoformulations, it was lower (20–30 cm), suggesting enhanced uptake by plants and less toxicity to non-target organisms such as fish and other aquatic organisms [66]. Thus, it can be said that encapsulation reduced the cytotoxicity of commercial mancozeb in the present study.

## 4. Conclusions

Mancozeb-loaded chitosan–gum acacia nanocomposites (nano CSGA-M) measuring 211.8–363.6 nm were synthesized via the ionic gelation and polyelectrolyte complexation methods. The TGA thermograph exhibited the higher thermal stability of the nanoform. In vitro, nano CSGA-M-1.5 at 1.5 ppm showed the greatest suppression against *Alternaria solani,* and is comparable to commercial mancozeb. At 1.0 and 1.5 ppm, nanoformulation showed full suppression against *Sclerotinia sclerotiorum*. In vivo disease control efficiency in pathogen-diseased plants using mancozeb-loaded nanocomposites was shown to be equivalent to commercial fungicide in pot conditions. For plant growth metrics, nano CSGA-M exhibited greater effectiveness, and also requires a growth co-relation study with advanced sophisticated techniques at molecular levels in large-scale field trials. The nanoformulation showed reduced toxicity to Vero cells in a Resazurin assay, and a soil microflora study can give more insight of non-target toxicity. With a minimum quantity and thus minimum leaching losses to non-target sites, encapsulation may help to combat the soil and water pollution menaces of commercial mancozeb. Thus, these nanoformulations may be explored as eco-friendly alternatives to harmful chemical pesticides for sustainable crop production.

## Figures and Tables

**Table 1:** Average size, PDI, and zeta potential of blank and loaded CSGA nanocomposites.

Nanoformulation	Size (nm)	PDI	Zeta Potential (mV)
CSGA Blank	211.8 ± 0.9	1.000 ± 0.7	−17.1 ± 0.8
CSGA-M-1.0	363.6 ± 0.7	0.969 ± 0.8	−6.99 ± 0.7

Mean ± SD; three replicates were retained from each treatment.

**Table 2:** In vitro mycelium inhibition efficacy of CSGA nanocomposites.

Fungus	Nanoformulation with Mancozeb (ppm)	CSGA NCs	CSGA NCs% Inhibition = dc − dt/dc × 100	Mancozeb (ppm)	Mancozeb	Mancozeb% Inhibition = dc − dt/dc × 100
		Fungus Diameter (mm)			Fungus Diameter (mm)	
*A. solani* (ITCC3640)	Blank NC, N 1.0	22.5 ± 0.6	65.4 ± 0.7c	-	-	-
	Loaded NC, NF 0.5	17.5 ± 0.7	73.1 ± 1.2bc	F 0.5	10.5 ± 0.5	83.8 ± 0.7b
	Loaded NC, NF 1.0	17.0 ± 0.5	75.8 ± 1.4bc	F 1.0	10 ± 0.8	84.6 ± 0b
	Loaded NC, NF 1.5	10.5 ± 0.8	83.8 ± 0.7b	F 1.5	10 ± 0.6	84.6 ± 0b
*S. sclerotiorum* (ITCC5492)	Blank NC, N 1.0	12.5 ± 0.8	63.2 ± 0.7c	-	-	-
	Loaded NC, NF 0.5	13.0 ± 0.9	59.9 ± 1.4c	F 0.5	10.5 ± 0.7	69.1 ± 0.7c
	Loaded NC, NF 1.0	0	100 ± 0a	F 1.0	0	100 ± 0a
	Loaded NC, NF 1.5	0	100 ± 0a	F 1.5	0	100 ± 0a

Each value is the average of three measurements. A *t*-test found that mean ± SD followed by the same letter in the treatment column are not statistically different from control at *p* ≤ 0.05.

**Table 3:** The impact of CSGA NCs on disease effectiveness against early blight and stem rot in potato plants grown in pots.

Treatments	*A. solani* (Potato Early Blight) P1		*S. sclerotiorum* (Potato Stem Rot) P2	
	Disease Severity (%)	DCE (%)	Disease Severity (%)	DCE (%)
Pure control C	10.5 ± 0.7	00.0 ± 0.0	13.5 ± 2.1	00.0 ± 0.0
Control CP	29.4 ± 1.6	00.0 ± 0.0c	27.4 ± 1.6	00.0 ± 0.0c
Fungicide F	08.0 ± 0.6	72.8 ± 1.1a	08.7 ± 1.0	68.2 ± 6.9a
Fungicide FP	09.9 ± 0.5	66.3 ± 2.2	12.9 ± 2.4	52.9 ± 3.4c
Blank NCs N1	08.8 ± 0.9	70.1 ± 1.4a	09.6 ± 1.4	65.0 ± 0.5b
Blank NCs N1P	10.3 ± 1.8	65.0 ± 0.6b	12.9 ± 2.3	52.9 ± 3.4c
Loaded NCs N1F	08.2 ± 0.7	72.1 ± 1.7b	09.4 ± 2.1	65.7 ± 4.2a
Loaded NCs N1FP	10.4 ± 2.6	64.6 ± 5.0a	10.9 ± 1.9	60.2 ± 1.4b

Each value is the average of three measurements. As verified by the *t*-test, the mean ± SD followed by the same letter in the treatment column are not statistically different at *p* ≤ 0.05.

## Data Availability

Not applicable.

## References

[1] Statista.com. https://www.statista.com/statistics/1080566/india-economic-contribution-of-potatoes/.

[2] Lal M., Sharma S., Yadav S., Kumar S., Mustafa Y. (2018). Management of late blight of potato. Potato-from Incas to All over the World.

[3] Suganthi D., Sharma O.P., Mohan G., Pruthi S., Kaur M. (2020). Importance of early blight of potato induced by (*Alternaria solani*) and its management. Biot. Res. Today.

[4] Chaudhari A.K., Singh A., Singh V.K., Dwivedy A.K., Das S., Ramsdam M.G., Dkhar M.S., Kayang H., Dubey N.K. (2020). Assessment of chitosan biopolymer encapsulated α-Terpineol against fungal, aflatoxin B1 (AFB1) and free radicals mediated deterioration of stored maize and possible mode of action. Food Chem..

[5] Divya K., Jisha M.S. (2018). Chitosan nanoparticles preparation and applications. Environ. Chem. Lett..

[6] Zielińska A., Carreiró F., Oliveira A.M., Neves A., Pires B., Venkatesh D.N., Durazzo A., Lucarini M., Eder P., Souto E.B. (2020). Polymeric nanoparticles: Production, characterization, toxicology and ecotoxicology. Molecules.

[7] Verma D.K., Malik R., Meena J., Rameshwari R. (2021). Synthesis, characterization and applications of chitosan based metallic nanoparticles: A review. J. Appl. Nat. Sci..

[8] Lupusoru R.V., Simion L., Sandu I., Pricop D.A., Chiriac A.P., Poroch V. (2017). Aging study of gold nanoparticles functionalized with chitosan in aqueous solutions. Chem. Rev..

[9] Anusuya S., Sathiyabama M. (2016). Effect of chitosan on growth, yield and curcumin content in turmeric under field condition. Biocatal. Agric. Biotechnol..

[10] Choudhary R.C., Kumaraswamy R.V., Kumari S., Sharma S.S., Pal A., Raliya R., Biswas P., Saharan V. (2017). Cu-chitosan nanoparticle boost defense responses and plant growth in maize (*Zea mays* L.). Sci. Rep..

[11] Chakraborty M., Hasanuzzaman M., Rahman M., Khan M., Rahman A., Bhowmik P., Mahmud N.U., Tanveer M., Islam T. (2020). Mechanism of plant growth promotion and disease suppression by chitosan biopolymer. Agriculture.

[12] Fortunati E., Balestra G.M. (2019). Lignocellulosic materials as novel carriers, also at nanoscale, of organic active principles for agri-food applications. Biomass, Biopolymer-Based Materials, and Bioenergy.

[13] Kaur P., Duhan J.S., Thakur R. (2018). Comparative pot studies of chitosan and chitosan-metal nanocomposites as nano-agrochemicals against fusarium wilt of chickpea (*Cicer arietinum* L.). Biocatal. Agric. Biotechnol..

[14] Morin-Crini N., Lichtfouse E., Torri G., Crini G. (2019). Applications of chitosan in food, pharmaceuticals, medicine, cosmetics, agriculture, textiles, pulp and paper, biotechnology, and environmental chemistry. Environ. Chem. Lett..

[15] Kim D.Y., Kadam A., Shinde S., Saratale R.G., Patra J., Ghodake G. (2018). Recent developments in nanotechnology transforming the agricultural sector: A transition replete with opportunities. J. Sci. Food Agric..

[16] Adisa I.O., Pullagurala V.L.R., Peralta-Videa J.R., Dimkpa C.O., Elmer W.H., Gardea-Torresdey J.L., White J.C. (2019). Recent advances in nano-enabled fertilizers and pesticides: A critical review of mechanisms of action. Environ. Sci. Nano.

[17] Menossi M., Casalongué C., Alvarez V.A. (2022). Bio-nanocomposites for Modern Agricultural Applications. Handbook of Consumer Nanoproducts.

[18] Avadi M.R., Sadeghi A.M., Mohamadpour Dounighi N., Dinarvand R., Atyabi F., Rafiee-Tehrani M. (2011). Ex vivo evaluation of insulin nanoparticles using chitosan and *Arabic gum*. ISRN Pharm..

[19] Chopra M., Kaur P., Bernela M., Thakur R. (2014). Surfactant assisted nisin loaded chitosan-carageenan nanocapsule synthesis for controlling food pathogens. Food Control.

[20] Campos E.V.R., de Oliveira J.L., da Silva C.M.G., Pascoli M., Pasquoto T., Lima R., Abhilash P.C., Fraceto L.F. (2015). Polymeric and solid lipid nanoparticles for sustained release of carbendazim and tebuconazole in agricultural applications. Sci. Rep..

[21] Bansal P., Kaur P., Kumar A., Duhan J.S. (2017). Microwave assisted quick synthesis method of silver nanoparticles using citrus hybrid “Kinnow” and its potential against early blight of tomato. Res. Crop.

[22] Bansal P., Kaur P., Surekha, Kumar A., Duhan J.S. (2017). Biogenesis of silver nanoparticles using *Aspergillus terreus*, its cytotoxicity and potential as therapeutic against human pathogens. Res. J. Pharm. Biol. Chem. Sci..

[23] Kaur P., Thakur R., Duhan J.S., Chaudhury A. (2018). Management of wilt disease of chickpea in vivo by silver nanoparticles biosynthesized by rhizospheric microflora of chickpea (*Cicer arietinum*). J. Chem. Technol. Biotechnol..

[24] Vurro M., Miguel-Rojas C., Pérez-de-Luque A. (2019). Safe nanotechnologies for increasing the effectiveness of environmentally friendly natural agrochemicals. Pest Manag. Sci..

[25] Shekhar S., Sharma S., Kumar A., Taneja A., Sharma B. (2021). The framework of nanopesticides: A paradigm in biodiversity. Mater. Adv..

[26] Kaur P., Thakur R., Barnela M., Chopra M., Manuja A., Chaudhury A. (2015). Synthesis, characterization and in vitro evaluation of cytotoxicity and antimicrobial activity of chitosan–metal nanocomposites. J. Chem. Technol. Biotechnol..

[27] Saharan V., Sharma G., Yadav M., Choudhary M.K., Sharma S.S., Pal A., Raliya R., Biswas P. (2015). Synthesis and in vitro antifungal efficacy of Cu–chitosan nanoparticles against pathogenic fungi of tomato. Int. J. Biol. Macromol..

[28] Nuruzzaman M.D., Rahman M.M., Liu Y., Naidu R. (2016). Nanoencapsulation, nano-guard for pesticides: A new window for safe application. Agric. Food Chem..

[29] Kumar S., Kumar D., Dilbaghi N. (2017). Preparation, characterization, and bio-efficacy evaluation of controlled release carbendazim-loaded polymeric nanoparticles. Environ. Sci. Pollut. Res..

[30] Duhan J.S., Kumar R., Kumar N., Kaur P., Nehra K., Duhan S. (2017). Nanotechnology: The new perspective in precision agriculture. Biotechnol. Rep..

[31] Kah M., Kookana R.S., Gogos A., Bucheli T.D. (2018). A critical evaluation of nanopesticides and nanofertilizers against their conventional analogues. Nat. Nanotechnol..

[32] Sarkar M.R., Rashid H.O., Rahman A., Kafi A., Hosen I., Rahman S., Khan M.N. (2022). Recent advances in nanomaterials based sustainable agriculture: An overview. Environ. Nanotechnol. Monit. Manag..

[33] Avadi M.R., Sadeghi A.M.M., Mohammadpour N., Abedin S., Atyabi F., Dinarvand R., Rafiee-Tehrani M. (2009). Preparation and characterization of insulin nanoparticles using chitosan and *Arabic gum* with ionic gelation method. Nanomed. Nanotechnol. Biol. Med..

[34] Manuja A., Kumar S., Dilbaghi N., Bhanjana G., Chopra M., Kaur H., Kumar R., Manuja B.K., Singh S.K., Yadav S.C. (2014). Quinapyramine sulfate-loaded sodium alginate nanoparticles show enhanced trypanocidal activity. Nanomedcine.

[35] Maluin F.N., Hussein M.Z., Yusof N.A., Fakurazi S., Idris A.S., Hilmi Z., Jeffery Daim L.D. (2019). Preparation of chitosan–hexaconazole nanoparticles as fungicide nanodelivery system for combating *Ganoderma* disease in oil palm. Molecules.

[36] Amin M.K., Boateng J.S. (2022). Enhancing stability and mucoadhesive properties of chitosan nanoparticles by surface modification with sodium alginate and polyethylene glycol for potential oral mucosa vaccine delivery. Mar. Drugs.

[37] Rodrigues S., da Costa A.M.R., Grenha A. (2012). Chitosan/carrageenan nanoparticles: Effect of cross-linking with tripolyphosphate and charge ratios. Carbohydr. Polym..

[38] Kumar S., Chauhan N., Gopal M., Kumar R., Dilbaghi N. (2015). Development and evaluation of alginate-chitosan nanocapsules for controlled release of acetamiprid. Int. J. Biol. Macromol..

[39] Giroud N., Dorge S., Trouvé G. (2010). Mechanism of thermal decomposition of a pesticide for safety concerns: Case of Mancoze. J. Hazard. Mater..

[40] Sun X., Liu C., Omer A.M., Yang L.Y., Ouyang X.K. (2019). Dual-layered pH-sensitive alginate/chitosan/kappa-carrageenan microbeads for colon-targeted release of 5-fluorouracil. Int. J. Biol. Macromol..

[41] Long J., Yu X., Xu E., Wu Z., Xu X., Jin Z., Jiao A., Jiao A. (2015). In situ synthesis of new magnetite chitosan/carrageenan nanocomposites by electrostatic interactions for protein delivery applications. Carbohydr. Polym..

[42] Piyakulawat P., Praphairaksit N., Chantarasiri N., Muangsin N. (2007). Preparation and evaluation of chitosan/carrageenan beads for controlled release of sodium diclofenac. AAPS PharmSciTech.

[43] Yien L., Zin N.M., Sarwar A., Katas H. (2012). Antifungal activity of chitosan nanoparticles and correlation with their physical properties. Int. J. Biomater..

[44] Ippólito S.D., Mendieta J.R., Terrile M.C., Tonón C.V., Mansilla A.Y., Colman S., Albertengo L., Rodríguez M.S., Casalongué C.A., Shalaby E. (2017). Chitosan as source for pesticide formulations. Biological Activities and Application of Marine Polysaccharides.

[45] Machado T.O., Beckers S.J., Fischer J., Müller B., Sayer C., de Araújo P.H., Landfester K., Wurm F.R. (2020). Bio-based lignin nanocarriers loaded with fungicides as a versatile platform for drug delivery in plants. Biomacromolecules.

[46] Vanti G.L., Masaphy S., Kurjogi M., Chakrasali S., Nargund V.B. (2020). Synthesis and application of chitosan-copper nanoparticles on damping off causing plant pathogenic fungi. Int. J. Biol. Macromol..

[47] Xu L., Cao L.D., Li F.M., Wang X.J., Huang Q.L. (2014). Utilization of chitosan-lactide copolymer nanoparticles as controlled release pesticide carrier for pyraclostrobin against *Colletotrichum gossypii* Southw. J. Dispers. Sci. Technol..

[48] Ilk S., Saglam N., Özgen M. (2017). Kaempferol loaded lecithin/chitosan nanoparticles: Preparation, characterization, and their potential applications as a sustainable antifungal agent. Artif. Cells Nanomed. Biotechnol..

[49] Maluin F.N., Hussein M.Z., Azah Yusof N., Fakurazi S., Idris A.S., Zainol Hilmi N.H., Jeffery Daim L.D. (2020). Chitosan-based agronanofungicides as a sustainable alternative in the basal stem rot disease management. J. Agric. Food Chem..

[50] Kheiri A., Jorf S.M., Malihipour A., Saremi H., Nikkhah M. (2017). Synthesis and characterization of chitosan nanoparticles and their effect on Fusarium head blight and oxidative activity in wheat. Int. J. Biol. Macromol..

[51] Sabry A.K.H., Salem H.A.N., Metwally H.M. (2021). Development of imidacloprid and indoxacarb formulations to nanoformulations and their efficacy against *Spodoptera littoralis* (Boisd). Bull. Natl. Res. Cent..

[52] Rico C.M., Majumdar S., Duarte-Gardea M., Peralta-Videa J.R., Gardea-Torresdey J.L. (2011). Interaction of nanoparticles with edible plants and their possible implications in the food chain. J. Agric. Food Chem..

[53] Ekner-Grzyb A., Chmielowska-Bąk J., Szczeszak A. (2021). Influence of GdVO_4_: Eu^3+^ nanocrystals on growth, germination, root cell viability and oxidative stress of wheat (*Triticum aestivum* L.) seedlings. Plants.

[54] Verma A., Prasher P., Sharma M., Kumar A., Mudila H., Kamel A.A.-E. (2021). Zinc oxide nanoparticles: Physiological and molecular responses in plants. Zinc-Based Nanostructures for Environmental and Agricultural Applications.

[55] Siddiqi K.S., Husen A. (2021). Plant response to silver nanoparticles: A critical review. Crit. Rev. Biotechnol..

[56] Mazumdar H., Ahmed G.U. (2011). Synthesis of silver nanoparticles and its adverse effect on seed germinations in *Oryza sativa*, *Vigna radiata* and *Brassica campestris*. Int. J. Adv. Biotechnol. Res..

[57] Yasmeen F., Razzaq A., Iqbal M.N., Jhanzab H.M. (2015). Effect of silver, copper and iron nanoparticles on wheat germination. Int. J. Biosci..

[58] González-García Y., Cadenas-Pliego G., Alpuche-Solís Á.G., Cabrera R.I., Juárez-Maldonado A. (2021). Carbon nanotubes decrease the negative impact of *Alternaria solani* in tomato crop. Nanomaterials.

[59] Awad A.A., Sweed A.A., Rady M.M., Majrashi A., Ali E.F. (2021). Rebalance the nutritional status and the productivity of high CaCo_3_-stressed sweet potato plants by foliar nourishment with zinc oxide nanoparticles and ascorbic acid. Agronomy.

[60] da Cruz T.N., Savassa S.M., Montanha G.S., Ishida J.K., de Almeida E., Tsai S.M., Lavres Junior J., de Carvalho H.W.P. (2019). A new glance on root-to-shoot in vivo zinc transport and time-dependent physiological effects of ZnSO_4_ and ZnO nanoparticles on plants. Sci. Rep..

[61] Bansal P., Kaur P., Duhan J.S. (2017). Biogenesis of silver nanoparticles using *Fusarium pallidoroseum* and its potential against human pathogens. Ann. Biol..

[62] Bansal P., Duhan J.S., Gahlawat S.K. (2014). Biogenesis of nanoparticles: A review. Afr. J. Biotechnol..

[63] Kumar R., Najda A., Duhan J.S., Kumar B., Chawla P., Klepacka J., Malawski S., Sadh P.K., Poonia A.K. (2022). Assessment of antifungal efficacy and release behavior of fungicide-loaded chitosan-carrageenan nanoparticles against phytopathogenic fungi. Polymers.

[64] Djiwanti S.R., Kaushik S., Prasad R. (2019). Nanopesticide: Future Application of Nanomaterials in Plant Protection. Plant Nanobionics. Nanotechnology in the Life Sciences.

[65] Shang Y., Hasan M., Ahammed G.J., Li M., Yin H., Zhou J. (2019). Applications of nanotechnology in plant growth and crop protection: A review. Molecules.

[66] Macedo D.F., Dourado S.M., Nunes E.S., Marques R.P., Moreto J.A. (2019). Controlled release of TBH herbicide encapsulated on Ca-ALG microparticles: Leaching and phytointoxication plants. Planta Daninha.

